# The Role of Dysfunctional Sleep Beliefs in Mediating the Outcomes of Web-Based Cognitive Behavioral Therapy for Insomnia in Community-Dwelling Older Adults: Protocol for a Single-Group, Nonrandomized Trial

**DOI:** 10.2196/32705

**Published:** 2022-12-27

**Authors:** Yvonne Kutzer, Lisa Whitehead, Eimear Quigley, Mandy Stanley

**Affiliations:** 1 School of Medical and Health Sciences Edith Cowan University Joondalup Australia; 2 School of Nursing and Midwifery Edith Cowan University Joondalup Australia; 3 School of Arts and Humanities Edith Cowan University Joondalup Australia

**Keywords:** older adults, insomnia, cognitive therapy, digital literacy, cognitive behavioral therapy for insomnia (CBT-I), online psychological intervention

## Abstract

**Background:**

Sleeping well is an essential part of good health. Older adult populations report a high rate of sleep problems, with recent studies suggesting that cognitive processes as well as behavioral and hyperarousal-related mechanisms could be important factors in the development and maintenance of insomnia. Individuals who have an asynchronous or uncoupled sleep pattern and sleep appraisal—those who complain about their sleep but do not have poor sleep quality, and vice versa—might show differences in subjective sleep and sleep perceptions and other characteristics that could impact their treatment outcomes following cognitive behavioral therapy for insomnia (CBT-I).

**Objective:**

The purpose of this protocol is to describe the rationale and methods for a nonrandomized, single-arm trial assessing objective and subjective sleep quality in community-dwelling older adults aged 60-80 years with synchronous sleep patterns and sleep appraisal compared to those in older adults with asynchronous sleep patterns and sleep appraisal. The trial will further examine the role of cognitive, behavioral, and hyperarousal processes in mediating the treatment outcomes of web-based CBT-I.

**Methods:**

This trial aims to recruit a sample of 60 participants, who will be assigned to 1 of 4 sleep groups based on their sleep pattern and sleep appraisal status: complaining good sleepers, complaining poor sleepers, noncomplaining good sleepers, and noncomplaining poor sleepers, respectively. The trial will be completed in 2 phases: phase 1 will assess objective sleep (measured via wrist actigraphy) and subjective (self-reported) sleep. Phase 2 will investigate the impact of a web-based CBT-I program on the sleep outcomes of individuals with uncoupled sleep compared to that of individuals without uncoupled sleep, as well as the mediators of CBT-I.

**Results:**

Recruitment began in March 2020, and the last participants were recruited by March 2021. A total of 65 participants completed phases 1 and 2. Data analysis for phase 1 was finished in December 2021, and data analysis for phase 2 was finalized in July 2022. The results for phase 1 were submitted for publication in March 2022, and those for phase 2 will be submitted by the end of December 2022.

**Conclusions:**

This trial will provide guidance on factors that contribute to the variability of sleep in older adults and their sleep outcomes following CBT-I. The outcomes of this study could be valuable for future research attempting to tailor CBT-I to individual needs.

**Trial Registration:**

Australian New Zealand Clinical Trials Registry ACTRN12619001509156; https://tinyurl.com/69hhdu2w

**International Registered Report Identifier (IRRID):**

DERR1-10.2196/32705

## Introduction

Sleep problems, such as insomnia, are highly prevalent in those older than 60 years of age [1], with age-related changes in sleep architecture appearing from middle age and continuing to remain relatively constant past the age of 60 years [2]. Insomnia is characterized by difficulties with sleep initiation, duration, or quality that are not due to a lack of opportunity to sleep and which results in daytime impairment [3]. It is classified as chronic when it occurs at least 3 times per week and persists for more than 3 months [4]. Furthermore, a diagnosis of insomnia consists of 2 components: sleep quality and sleep appraisal. Sleep quality can be good or poor and sleep appraisal can be characterized by the presence or absence of a sleep complaint. These 2 factors have been described as being independent of each other [5], and in about 25%-35% of individuals, sleep quality and sleep appraisal are asynchronous and uncoupled [6]. It has been proposed that such uncoupling of sleep quality and sleep complaints may be prevalent among older adults [7,8]. In their seminal study examining poor sleepers that do not complain of insomnia, Fichten et al [9] first observed the asynchrony of sleep complaints and sleep patterns in older individuals. They reported that daytime impairment, assessed as sleepiness and daytime fatigue was higher in high-distress poor sleepers than in low-distress poor sleepers, and that low-distress poor sleepers experienced the same levels of daytime impairment as good sleepers.

The distinction between high- and low-distress, or asynchronous, good and poor sleepers could also be relevant for the treatment of sleep problems, as it could signify differences in how individuals respond to insomnia treatment. Cognitive behavioral therapy for insomnia (CBT-I) is the recommended first-line treatment method to address insomnia in any age group [10]. It contains several core components such as psychoeducation, cognitive therapy, and behavior therapy [11,12]. However, despite a widespread recommendation for the use of CBT in the treatment of insomnia, the provision of pharmacotherapy remains the dominant approach to address sleep problems in older adults. This is in part due to a lack of trained clinicians [13]. The pharmaceutical treatment of insomnia can lead to polypharmacy, the concurrent use of multiple medicines, which is an identified issue for older individuals [14]. Polypharmacy can result in interaction effects or side effects, for example, an increased fall risk, in older adults [15,16]. CBT-I’s effectiveness and safety on the other hand have been well established, demonstrating moderate to large effects on improving sleep continuity [17]. CBT-I is also an effective treatment option for older adults, with recent research suggesting that even brief interventions can improve sleep onset latency, wake-after-sleep onset, sleep efficiency, and sleep quality [18,19], making it a viable alternative for older individuals at risk of polypharmacy.

The exact mechanisms by which such improvements are achieved remain unknown, and it is not fully understood why some individuals respond to CBT-I and others do not. It is estimated that 20%-30% of individuals with insomnia do not have a treatment response to CBT-I [20]. Cognitive processes are important factors in the development and maintenance of insomnia [5,20-27], particularly in older adults [28-30]. Discrepancies in subjective sleep perception and objectively measured sleep are also common [31]. In order to scrutinize sleep quality in older adults and the role that dysfunctional beliefs and other cognitive or behavioral factors play in mediating the outcomes of CBT-I, this study will examine both subjective and objective sleep quality in older adults aged 60-80 years in Western Australia. The age category of 60 years and older has been selected as it is the United Nations’ agreed cutoff for the classification of older persons in high-income nations [32]. The upper cutoff of 80 years was chosen based on an American Academy of Sleep Medicine report highlighting that the sleep variability in adults aged 80 years and older is either significantly higher or significantly lower than that in adults under 65 years [33,34]. Consequently, adults aged older than 80 years will be excluded.

The concurrent measurement of objective and subjective sleep quality will be used to determine the prevalence of individuals with asynchronous sleep patterns and sleep appraisal in this sample (phase 1). Phase 2 will assess the impact of a web-based CBT-I program on the sleep outcomes of individuals with synchronous sleep patterns and sleep appraisal and those with asynchronous sleep patterns and sleep appraisal. It is anticipated that complaining good sleepers and complaining poor sleepers will show improved sleep outcomes following CBT-I, compared with noncomplaining good and poor sleepers. We further hypothesize that individuals with a sleep complaint but without poor sleep quality (complaining good sleepers) experience higher levels of dysfunctional sleep cognitions and distress than persons with poor sleep in the absence of a sleep complaint (noncomplaining poor sleepers). We expect that additional factors such as hyperarousal [35], sleep effort [36], sleep-related self-efficacy [37], sleep locus of control [38], sleep-related behaviors [39], and chronotype [40] mediate the relationship between web-based CBT-I and sleep outcomes in older adults; therefore, these processes will also be examined.

Since our planned study is likely to generate a considerable amount of data, we aim to reduce publication bias and improve the reproducibility of our research by publishing this research protocol.

## Methods

### Ethics Approval

Ethical approval for this study has been granted by the Edith Cowan University Human Research Ethics Committee (reference STREAM 22000). All participants will provide written informed consent.

### Recruitment

Older adults with and those without self-reported poor sleep will be recruited from the community in Western Australia, with advertisements being displayed on the Sleep Health Foundation website and social media sites such as Facebook, as well as in community centers and senior citizen organizations. Participants are eligible to take part if they are aged 60-80 years and ordinarily reside in Western Australia, if they have not been diagnosed with an existing sleep disorder other than insomnia, if they have not been diagnosed with a severe psychiatric or cognitive disorder, if they have not engaged in regular shift work in the past year, and if they have not been diagnosed with epilepsy or are at high risk of falling.

Screening for the presence of obstructive sleep apnea, which is common among older adults, will be conducted using the STOP-Bang (snoring, tiredness, observed apnea, high BP, BMI, age, neck circumference, and male gender) questionnaire, a brief self-report instrument [41]. The STOP-Bang questionnaire has demonstrated a high sensitivity of 84% in detecting obstructive sleep apnea [41]. Participants that score 0 to 2 on the instrument are considered unlikely to present with moderate to severe sleep apnea. Participants who took sleep medication were not excluded, as CBT-I has shown to be effective in those who take sleep medication [12]. However, the type, dosage, and frequency of any medication taken by participants were recorded as part of the demographic assessment.

### Design

Study participants will be required to complete a web-based questionnaire battery at baseline and following the intervention. It is estimated that it will take approximately 20-25 minutes to complete the questionnaire battery. Sleep-related dysfunctional beliefs will be assessed using the Dysfunctional Beliefs and Attitudes about Sleep (DBAS-16) scale [42]. The DBAS-16 was developed to examine sleep-related cognitions and dysfunctional beliefs. Items such as “I am worried that I may lose control over my abilities to sleep” are scored on a 10-point Likert scale ranging from 0 to 10. Like the original 30-item version, the DBAS-16 has been found to reliably discriminate between self-reported good and poor sleepers in older adult populations with adequate internal consistency (Cronbach α=.79 for research samples). A DBAS score of 4 or above is indicative of a clinically significant level of dysfunctional beliefs around sleep [21].

The Pittsburgh Sleep Quality Index [43] consists of 19 items and examines sleep quality and disturbances over the past month. The first 4 questions are open, whereas items 5 to 19 are rated on a 4-point Likert scale. Individual item scores from the 7 sections are calculated first, and those 7 component scores are subsequently added up to calculate the global score ranging from 0 to 21. A sleep score of >5 is indicative of poor sleep quality. The Pittsburgh Sleep Quality Index measures sleep quality in a broader sense than just insomnia severity and has good internal consistency (Cronbach α=.83).

The Insomnia Severity Index [44,45] is a brief self-report questionnaire used to measure the severity of insomnia, impairment of daytime functioning, and worry about sleep over the past 2 weeks. It examines both nighttime and daytime components of insomnia and is increasingly used in research as an instrument to examine treatment response. The Insomnia Severity Index has been shown to have excellent internal consistency in community samples (Cronbach α=.90).

The Glasgow Sleep Effort Scale [36] reviews sleep effort. Sleep effort is a concept that explains a person’s voluntary effort to control a partially involuntary process, namely falling asleep. The Glasgow Sleep Effort Scale consists of 7 items, for example, “I put too much effort into sleeping when it should come naturally,” rated on a 3-point scale (“very much”, “to some extent”, and “not at all”). Internal consistency was reported as fair, with Cronbach α=.77 [36].

The Self-Efficacy for Sleep Scale [37] assesses the “belief people have in their ability to participate in certain behaviors needed to promote their health” [46,47]. The Self-Efficacy for Sleep Scale is a 9-item measure of how confident an individual is to carry out a variety of behaviors related to sleep, for example, “fall asleep at night in less than 30 minutes.” Items are scored on a 5-point scale, with higher scores being indicative of increased sleep self-efficacy. The SES has fair internal consistency (Cronbach α=.71) [48].

The Sleep Locus of Control Scale [38] is an 8-item measure that examines the internal sleep locus of control, the belief that sleep outcomes are dependent on one’s behavior on a 6-point Likert scale. The Sleep Locus of Control Scale contains 2 subscales, internal locus of control and chance sleep locus, and is reported to have internal consistency of Cronbach α=.72 (internal locus of control) and .59 (chance sleep locus) [38].

Hyperarousal will be assessed using the Pre-Sleep Arousal Scale (PSAS) [35]. The PSAS measures cognitive and somatic symptoms of arousal before falling asleep using 2 subscales. The 2 subscales each consist of 8 items. The items are rated using a 5-point Likert scale, with scores ranging from 1 to 5. The internal consistency of the PSAS has been reported as Cronbach α=.67 for the cognitive and .84 for the somatic subscales in normal sleepers and .76 and .81, respectively, for individuals with insomnia.

The Morningness-Eveningness Questionnaire [40] assesses chronotype. The 19-item scale was developed to examine individuals’ alertness at specific times of the day. The 19-item scale examines preferences in sleep and wake times and what times of the day subjects feel most alert. The Morningness-Eveningness Questionnaire has shown sufficient internal consistency for the original (Cronbach α=.82) as well as for the reduced version [49].

The Sleep-Related Behaviors Questionnaire [39] is a 32-item instrument that assesses safety behaviors relating to sleep; for example, “I miss or cancel appointments (daytime or evening).” Items are rated on a 5-point scale ranging from 0 (meaning “almost never”) to 4 (meaning almost always”). The questionnaire has high internal consistency (Cronbach α=.92).

Participants will wear an actigraph and complete a sleep diary over 96 hours (72 hours plus an additional 24 hours to account for nonadherence wear time). Actigraphic sleep assessment will assess sleep onset latency, wake-after-sleep onset, total sleep time, and the number of nighttime awakenings. The Actigraph model wGT3X-BT activity monitor (ActiGraph) will be used. Actigraphy has been recognized by the American Academy of Sleep Medicine as an appropriate method for assessing sleep. However, when compared with polysomnography, actigraphy has lower accuracy in detecting wake periods; especially in individuals with poor sleep quality [50]. The actigraphy data will be manually scored along with a sleep diary. This process was recommended by Boyne et al [51] to optimize agreement with polysomnography-measured sleep-wake. Additionally, the Choi algorithm will be used to differentiate between wear and nonwear times, which more accurately estimates time spent sedentary than other algorithms [52]. The sleep diary is a modified version of the Consensus Sleep Diary [53], which, in addition to the questions covered by the Consensus Sleep Diary, records the total duration of all daytime naps, how many times the study participant got up during the night, what time they had planned to wake up at, and whether they took any sleep medication.

Participants will be grouped into 4 sleep groups: noncomplaining good sleepers, complaining good sleepers, noncomplaining poor sleepers, and complaining poor sleepers. Participants will be grouped based on their actigraphy results and sleep complaint status (complaining versus noncomplaining sleepers), with participants appraised as poor sleepers if their sleep onset latency or wake-after-sleep onset is ≥31 minutes, 3 times or more during the recording period (as per actigraph recording). A participant will be considered a complaining sleeper if they report having had a sleep problem (eg, trouble falling asleep) for a minimum of 6 months at baseline data collection [5,54].

### Procedure

Participants who express an interest in participating will be contacted to make an appointment for a screening call. During the screening call, potential participants will be provided with information about the study and will be assessed against the eligibility criteria. Once a potential participant is confirmed to be eligible, they will complete, sign, and return the consent form. Upon study commencement, potential participants will be provided with an individual web-based link to the Qualtrics questionnaire battery (Qualtrics XM Platform software, Qualtrics). Once the questionnaire has been completed, actigraphs will be posted to participants using a courier service. Delivery and collection times will be arranged before dispatch at a time convenient for the participant. Where courier services are not available, the sleep watches will be posted by registered express mail, including a prepaid self-addressed return envelope. Study participants will be required to wear an actigraph on their nondominant wrist for 96 hours, except when they take a bath, have a shower, go swimming, or undergo any other activity that would result in them submerging the actigraph in water. They will also be instructed to record the exact time when they remove the actigraph and, subsequently, when they reattach the actigraph to their wrist in their sleep diary. This allows for accurate matching of the actigraph-estimated wear and nonwear times with the sleep diary. The sleep diary will be completed concurrently to record bedtimes, sleep and wake times, frequency and duration of nocturnal awakenings, rise times, daytime naps, and nonwear times, and will be returned with the actigraph after the measurement has been completed. A printed copy of the sleep diary will allow the participant to keep the diary on their bedside table so the relevant sections can be completed upon going to bed and waking up in the morning.

**Figure 1:**
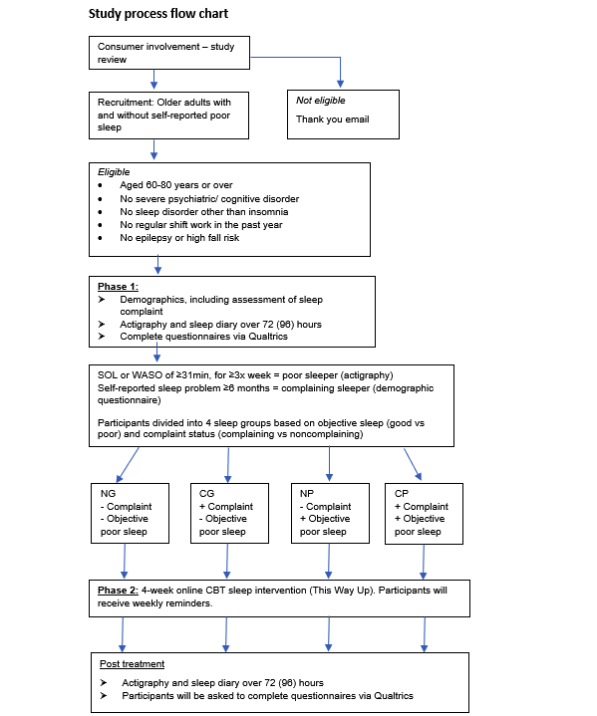
Study process flowchart. CBT: cognitive behavioral therapy; CG: complaining good; CP: complaining poor; NG: noncomplaining good; NP: noncomplaining poor; SOL: sleep onset latency; WASO: wake-after-sleep onset.

Following the baseline assessment, including the recording of demographic information and sleep complaints, the administration of the questionnaires, and actigraphy measurement, all groups will receive identical instructions for the web-based CBT-I program. Participants will be required to complete the 4 sessions plus homework of the program within 4 weeks. Participants will receive weekly email reminders from the principal investigator to confirm the completion of each of the 4 CBT-I modules. Once the completion of the CBT-I program has been confirmed with the participant, actigraphy, sleep diary, and questionnaire measurements will be repeated posttreatment. The study process is shown in Figure 1.

### Description of Intervention

There are a variety of web-based CBT-I courses, mostly developed in the United Kingdom or the United States. One exception is the “This Way Up—Managing Insomnia” program [55,56]. This treatment course was created by a team of clinical psychologists and psychiatrists at the Clinical Research Unit for Anxiety and Depression, which is run by the University of New South Wales and St Vincent’s Hospital in Sydney. The free self-help program consists of 4 lessons in a comic-based format. Lesson 1 provides background knowledge about sleep and insomnia and which factors are conducive to good sleep and which ones are related to insomnia (sleep hygiene). Lessons 2 and 3 address the management of thoughts and behaviors that interfere with sleep (sleep restriction, stimulus control, and cognitive therapy). The sleep restriction protocol aims at decreasing nighttime awakenings by limiting the time a person spends in bed [57]. Initially, the prescribed time in bed corresponds with the average total sleep time as measured in the week prior to initiating the sleep restriction protocol, with a minimum time in bed of 5.5 hours. Time in bed is then gradually increased until the amount of nighttime sleep achieved is sufficient for individual requirements. Lesson 4 focuses on relaxation techniques. The program cannot be individually tailored to the patient’s specific sleep difficulties but provides broad topic coverage of the most common factors present in insomnia (eg, waking too early), and program participants are advised that not all themes covered may be relevant to their circumstances.

Each lesson takes about 20 minutes to complete, with additional compulsory homework. A unit only registers as completed once the homework has been downloaded. It is recommended that a user completes 1 lesson every 1-2 weeks. However, the system will not grant access for less than 5 days between modules. Reminders are emailed or sent by text message automatically once a new session becomes available, and there is also the option to set a web-based appointment for the next course module. In addition to the course unit and homework, participants are required to complete a sleep diary. Upon completion of the course, an extra 12 months’ access is made available for additional practice. The program is available only to individuals who live in Australia.

The program’s easy-to-access platform makes it suitable even for individuals with low eHealth literacy. This could be important when providing web-based CBT-I to older populations, even though older adults are increasingly familiar with using the internet and social media. A recent report exploring the attitudes and behaviors of adults aged 70 years and older pointed out that 99% of individuals have searched for information on the internet, 65% of them post on or read from social media, and 82% of them who are on social media use Facebook [46].

Participants with moderate to extremely severe total scores on any scale of the DASS-21 (Depression, Anxiety, and Stress Scale-21) will receive a letter outlining the results and will be encouraged to discuss these with their health care professionals. This is considered an advisable measure of duty of care. However, while the DASS-21 measures the emotional states of anxiety, stress, and depression over the previous week, it is based on a dimensional rather than a categorical conception of disorder and should not be used as a diagnostic tool [58]. Participants with ongoing or severe insomnia, stress, anxiety, or depression symptoms will be advised to contact the university’s Psychological Services Centre or community services such as Lifeline.

Consideration will also be given to the delivery of a sleep restriction module as part of the web-based CBT-I. Any contraindications to CBT-I usually arise from the use of the sleep restriction element and occur when an individual has epilepsy, bipolar disorder, or is at high risk of falling, and can also exacerbate symptoms in those with excessive daytime sleepiness, for example, in individuals with sleep apnea [59]. Participants will be screened for obstructive sleep apnea risk using the STOP-Bang questionnaire and will be asked if they have ever been diagnosed with epilepsy or severe psychiatric (eg, bipolar disorder) or cognitive impairment (eg, mild cognitive impairment). Participants will also be questioned about their fall risk, including queries about whether they have had a fall in the past year, feel unsteady when standing or walking, or if they are worried about falling. Participants with epilepsy, bipolar disorder, cognitive impairment, a sleep apnea diagnosis, or a high fall risk will be excluded from the study. Individuals with any other psychiatric disorder (eg, borderline personality disorder) will be excluded depending on their active symptomatology and current management of the condition, following consultation with the clinical psychologist on the research team. Cognitive disorders and personality disorders will be assessed through self-report.

### Statistical Analysis

An a priori power analysis using the program G*Power was performed to assess whether the study will have enough power to detect significant differences in sleep outcomes between the study groups [60]. We calculated that a sample size of 40 would be sufficient to detect this size effect. In order to account for attrition rates and missing data, a minimum of 60 participants will be recruited for the study.

Since sleep complaint status (sleep problem, eg, initial insomnia, for a minimum of 6 months) is used to classify participants as complaining or noncomplaining sleepers, multivariate ANOVA will be conducted separately to examine subjective and objective sleep quality for the 4 sleep groups. The multivariate ANOVA enables the analysis of multiple continuous dependent variables simultaneously (eg, sleep onset latency, wake-after-sleep onset, total sleep time, and the number of nighttime awakenings). In addition, a mediation analysis will be performed to determine whether sleep improvements following web-based CBT-I are the result of changes in sleep-related beliefs or other factors in older adults living in the community. Mediation analysis will be carried out using the bootstrapping method in the Hayes Process Macro for mediation, moderation, and conditional analysis for SPSS (version 28.0; IBM Corp) [61,62].

Actigraphy measures will be scored using the Cole-Kripke algorithm, which is suitable for use with older populations [63] and using ActiLife software (ActiLife 6 software, Version 6.13.1, ActiGraph). The autoscored data will be manually compared with the sleep diary to determine any incongruence between the actigraph measurements and the sleep diary.

## Results

Study recruitment was completed in March 2021, with a total of 65 participants. In addition, a feasibility study to assess whether the study format was acceptable for participants aged 60-80 years was conducted from October 2019 to 2020.

Data analysis for phase 1 was finished in December 2021, and data analysis for phase 2 was finalized in July 2022. The results for phase 1 have been submitted for publication in March 2022, and those for phase 2 will be submitted by the end of December 2022.

## Discussion

### Expected Findings

We anticipate that our study will highlight the variability of sleep patterns in older adults and the factors that impact their sleep outcomes following CBT-I. Since the mid-1990s, the need to examine the asynchronous sleep pattern characterized by a mismatch between objective sleep quality and subjective sleep complaint has been highlighted in the literature [7-9,64]. Research to date has indicated that higher levels of distress and maladaptive cognitions, particularly as displayed by complaining sleepers with a subjective sleep complaint, show a stronger correlation with daytime impairment than with objective sleep disruption, and that individuals with subjectively poor sleep report lower sleep quality even if their objective sleep is within acceptable parameters [65]. In addition, the prevalence of complaining good sleepers appears to be high in older adults [26].

The COVID-19 pandemic and its associated impact on mental and physical health outcomes add an additional dimension to the need to investigate sleep health and the role sleep perceptions play in insomnia. Sella et al [66] stressed in their recent paper that during the COVID-19 pandemic, changes in self-reported sleep quality in older adults were predominantly associated with changes in dysfunctional sleep beliefs. Furthermore, the pandemic has brought the need for web-based health services, such as digital CBT-I, to the forefront. Recent systematic reviews suggest that CBT-I reduces dysfunctional sleep beliefs, which contributes to the improvement of insomnia symptoms [27,67].

In our study, we will use actigraphy to measure objective sleep outcomes. Actigraphic sleep assessment can provide an accurate estimate of sleep and wake patterns, even when compared with the gold standard of sleep measurement, polysomnography. It has been recommended for the estimation of sleep parameters in adults with insomnia [68], but the limitation of actigraphy is that it underestimates total sleep time [69]. However, research has also indicated that actigraphy is sensitive in detecting treatment effects [70], making it suitable for assessing the sleep outcomes following CBT-I.

This study will add to the knowledge base regarding CBT-I outcomes in an older adult population and will help ascertain whether asynchronous sleep patterns and sleep appraisal as well as associated maladaptive sleep beliefs, high arousal, and other cognitive factors play a role in this process.

### Conclusions

It is crucial to examine sleep quality in older adults as this age group shows a high rate of sleep disturbances. The relatively high prevalence of older adults who are complaining good sleepers is of particular concern. Individuals with a subjective sleep complaint often report worse outcomes regardless of how well they sleep, which suggests that insomnia might not arise from sleep deprivation but could be associated with how sleep quality is perceived by the individual.

To address these concerns and examine the prevalence of uncoupled sleepers specifically in the Western Australian context, this study will assess subjective and objective sleep quality in community-dwelling older adults aged 60-80 years and examine the role of cognitive processes, such as dysfunctional sleep beliefs, in mediating the treatment outcomes of a digital cognitive behavioral therapy program for insomnia.

## Abbreviations

CBT-I: cognitive behavioral therapy for insomnia
DASS-21: Depression, Anxiety and Stress Scale-21
DBAS-16: Dysfunctional Beliefs and Attitudes about Sleep
PSAS: Pre-Sleep Arousal Scale
STOP-Bang: snoring, tiredness, observed apnea, high BP, BMI, age, neck circumference, and male gender
